# Design of static synchronous series compensator based damping controller employing invasive weed optimization algorithm

**DOI:** 10.1186/2193-1801-3-394

**Published:** 2014-07-30

**Authors:** Ashik Ahmed, Rasheduzzaman Al-Amin, Ruhul Amin

**Affiliations:** EEE Department, Islamic University of Technology, Board Bazar, Bangladesh; ETE Department, International Islamic University, Chittagong, Bangladesh; EEE Department, Bangladesh University of Business and Technology, Dhaka, Bangladesh

**Keywords:** Invasive weed optimization, Static synchronous series compensator, Single machine infinite bus power system, Power oscillation damping (POD), Integral of time absolute error

## Abstract

This paper proposes designing of Static Synchronous Series Compensator (SSSC) based damping controller to enhance the stability of a Single Machine Infinite Bus (SMIB) system by means of Invasive Weed Optimization (IWO) technique. Conventional PI controller is used as the SSSC damping controller which takes rotor speed deviation as the input. The damping controller parameters are tuned based on time integral of absolute error based cost function using IWO. Performance of IWO based controller is compared to that of Particle Swarm Optimization (PSO) based controller. Time domain based simulation results are presented and performance of the controllers under different loading conditions and fault scenarios is studied in order to illustrate the effectiveness of the IWO based design approach.

## Introduction

Power oscillation is a familiar dynamic fact that arises in power system when subjected to disturbance. If adequate damping is not provided, these unwanted oscillations may survive and cause system separation (Kundur 1994). Power system stabilizers (PSS) came into forth with the idea of damping these oscillations by injecting supplementary excitation control signal and increase the stability of the power system. However, PSS were found responsible for causing significant variations in voltage level which may lead to power system instability during the time of three phase faults.

Flexible ac transmission systems (FACTS) utilize power electronic based fast switching devices which can control power flow in the lines and improve stability (Padiyar 2007). FACTS devices are considered as the prominent ones among many effective means to improve operation of power system, increase power transfer capacity etc. Series capacitive compensation method has been employed to remove significant portion of the reactive line impedance and hence improve the amount of transmittable power under dynamic conditions. Static Synchronous Series Compensator is a voltage source converter based FACTS device which is connected in series with the transmission line (Gyugi et al. 1997).

SSSC injects a controllable and almost sinusoidal voltage which remains in series with the transmission network (Hingorani & Gyugi 2000). The injected voltage source imposes virtual reactance in the line which in turn controls the power flow of the transmission line. This control of line power flow is independent of the magnitude of the line current (Hingorani & Gyugi 2000). The ability of SSSC to operate in both inductive and capacitive mode makes it very efficient in controlling the power flow in the system. In either case the injected voltage remains in quadrature with the line current and therefore acts as capacitive or inductive reactance in series with the transmission line. Besides controlling line power flow, SSSC offers good response time with perfectly smooth transition from (+ve) positive to (−ve) negative power through zero voltage injection. Unlike other series compensating devices, SSSC does not run the risk of getting into classical resonance issues at fundamental frequency operation because of the fact that for every practical scenarios line inductance (L) is essentially regulated by injected compensating voltage produced (Acha et al. 2004). Aside from controlling the power flow of the line, SSSC can be utilized as a Power Oscillation damping (POD) device through modulation of series reactive power compensation (Zhang et al. 2006). The energy storage ability of SSSC can enhance the effectiveness of POD by absorbing or injecting real power into the transmission line.

The attractive features and effectiveness of SSSC has made its use widespread in very short period. The SSSC based damping controller design has been handled differently in recent literatures. Time optimal control theory has been employed to design a SSSC based damping controller in (Pandey & Singh 2008). A simplified two area system is considered and the linearized power system model is used. The drawback of this work is that the solution of the Riccati equation is time consuming and large matrix manipulations are required to obtain the desired result. Three different operating modes of SSSC are identified in (David & Venkataramanan 2007) and the controller design problem is handled by frequency domain based loop shaping technique. The results presented shows that the response time taken by the proposed controllers is quite large. Tuning of the SSSC damping controller is performed by real coded genetic algorithm (RCGA) in (Swain et al. 2011). An integral time absolute error based objective function is selected and deviation in the rotor speed from the synchronous speed is referred as the error. Like genetic algorithm, RCGA, too, has a tendency to get stuck at a local minimum of the solution space. A differential evolution (DE) based approach is proposed in (Swain et al. 2013). Built-in Simulink blocks are used for the analysis which may not allow much freedom for the user to work with. No information is provided regarding the convergence scenario of the proposed algorithm which is one of the major criteria to evaluate an optimization algorithm. Similar drawbacks are observed in (Swain et al. 2012). Adaptive Neuro-fuzzy inference system (ANFIS) is employed to design the damping controller of SSSC in a multi-machine power system network (Murali & Rajaram 2010). The deviation in line power is taken as the error signal for the controller and the output is the SSSC injected voltage magnitude. Self- tuning PID controller is utilized in (Therattil & Panda 2011) to damp out electromechanical oscillations. The responses show that it takes around 5 seconds to completely suppress the oscillations. Nonlinear adaptive control technique is proposed for the SSSC damping controller design (Gu et al. 2010). Like any adaptive control algorithm, it takes a healthy computation time to get the required control effort. Design of SSSC damping controller is modeled as a multi-objective optimization problem and solved using Particle Swarm Optimization (PSO) algorithm in (Ajami & Armaghan 2010). Different loading scenarios are considered to show the effectiveness of the proposed method. Nonlinear feedback linearization control technique is employed in (Ghaisari & Bakhshai 2005) where the study system dynamic model is represented as a multi-input multi-output system. Although the paper describes the need of zero-dynamic study for the stability of overall system, it is not explained for the study system.

Invasive Weed Optimization (IWO) is a recent meta-heuristic search algorithm which is inspired from the weed colonizing technique (Mehrabian & Lucas 2006). IWO was tested for different multi-dimensional benchmark systems and the performance is compared with other efficient search algorithms. Performance of IWO was found superior to Genetic Algorithm, Simulated Annealing, Particle Swarm Optimization, Memetic Algorithm and Shuffled Frog Leaping algorithm. From then on IWO has found numerous applications in diversified field of engineering and science. IWO is efficiently applied for optimizing and tuning of a robust controller (Mehrabian & Lucas 2006), designing an E-shaped MIMO antenna (Mallahzadeh et al. 2009), optimal positioning of piezoelectric actuators (Mehrabian & Yousefi-Koma 2007), studying the electricity market dynamics (Ardakani et al. 2008), designing the encoding sequences for DNA computing (Zhang et al. 2009), and developing a recommender system (Rad & Lucas 2007).

This paper utilizes the superior performance of IWO to find out the optimal parameter of SSSC damping controller in an SMIB system. A time-domain based objective function is chosen which considers deviation in the rotor speed as error signal and the job of the optimizer is to minimize the error in the quickest possible time. The performance of IWO is then compared to that of PSO based controller.

The paper is organized as follows: Mathematical model discusses the system investigated and SSSC structure, Invasive weed optimization discusses Invasive Weed Optimization Algorithm. The simulation results are presented and discussed in Simulation results. Finally, the conclusions are given in Conclusion.

## Mathematical model

### System model and SSSC structure

**Figure 1:**
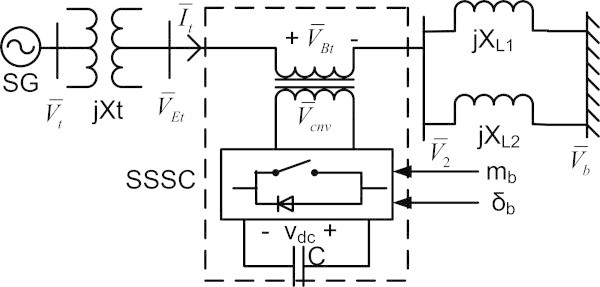
**Single machine infinite bus power system with SSSC.**

Consider a single machine infinite bus system with static synchronous series compensator in series with transmission line as depicted in Figure 1.

The SSSC comprising of a voltage source converter, a series injection transformer and a dc-storage capacitor is indicated in a dotted block. The SSSC injects  into the transmission line which can be modulated for control of power flow in the line. The control variables are the modulation index m_b_ and phase angle δ_b_ of the voltage source converter.

The synchronous generator (SG) is represented by the 3rd order non-linear mathematical model with IEEE- ST1 type exciter (Anderson & Fouad 1977). The SG terminal voltage is represented by . The transformer with reactance X_t_ steps up the generator voltage to . The SSSC series injection transformer boosts up the voltage  to . The relation between the fundamental frequency component of  and the dc capacitor voltage V_dc_ is (Therond 1999):
1

 and  are the mid-bus and infinite bus voltage, respectively. XL1 and XL2 represent reactance of the parallel transmission lines. The infinite bus voltage is taken as the reference with a constant magnitude. The swing equation of the SG is given as:
23

The q-axis transient voltage dynamics is given as:
4

The exciter dynamics is represented as:
5

Dynamics of SSSC dc link voltage is:
6

The algebraic quantities appearing in equations (2) - (6) are given as follows:
78

9Applying KVL at different nodes of Figure 1:
1011

Combining (8) and (9) the following is obtained:
12

Equation (11) can be re-written in terms of d and q components:
13

The d and q components of the booster transformer voltage V_Btd_ and V_Btq_ can be expressed as:
1415

The d and q components of the infinite bus voltage can be written as:
1617

Equating the R.H.S. of equations (12) and (13) and solving for I_td_ and I_tq_ using the expressions from (14)-(17):
1819

where,


### SSSC damping control

**Figure 2:**
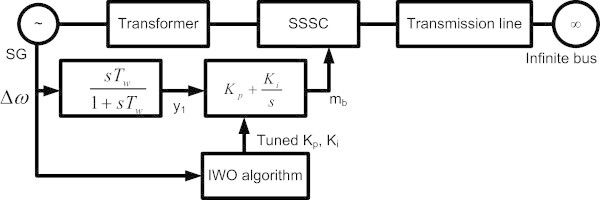
**SSSC damping controller connected to the SMIB system.**

The damping controller configuration of the SSSC is depicted in Figure 2. Among the two control signals available, the modulation index m_b_ has been used in this paper as it shows greater controllability over δ_b_ (Therattil & Panda 2011).

The rotor speed deviation Δω is achieved by introducing different dynamic scenarios for different loading conditions. The system is initialized each time there is any change in operating condition. The rotor speed deviation is taken as the input to the PI controller through a wash-out block. The same speed deviation signal is passed to the IWO optimizer which calculates an objective function and tries to obtain an optimal solution to the problem at hand by minimizing the deviation in the speed signal. The IWO optimizer then sends the optimized parameter set (in this case K_p_ and K_i_) to the controller block and the controller output gives the desired magnitude of modulation index (m_b_) in order to improve the system damping. The optimizer is applied for a certain fault case and loading condition.

With the introduction of the PI controller along with a wash-out block, the new dynamic equations are:
2021

### Objective function

The design of the PI based SSSC damping controller is formulated as a single objective constrained optimization problem and solved by IWO technique. The objective function considered in this paper is:


where, tsim is the total simulation time. For a certain parameter set, the objective here is to minimize the J value subject to the following constraints:


## Invasive weed optimization

Invasive Weed Optimization is a bio-inspired numerical stochastic optimization algorithm that simply simulates natural behavior of weeds in colonizing and finding suitable place for growth and reproduction. Some of the distinctive properties of IWO in comparison with other evolutionary algorithms are the way of reproduction, spatial dispersal, and competitive exclusion (Mehrabian & Lucas 2006).

### The IWO process is summarized as follows

#### Initialize a random population

To start with, a random population set is defined over the allocated search space. The allocated search space is generally confined to the constrained boundaries.

#### Reproduction

Each randomly produced seeds are tested on the objective function to find out their individual fitness to achieve a certain target. These seeds are now allowed to reproduce depending on their own fitness in a linear fashion, i.e., none of the seeds are excluded from the regeneration phase. Each seed has a chance to reproduce and the reproduction rate varies from the maximum to the minimum for the best to the worst fit seed, respectively.

#### Spatial dispersal

The reproduced seeds are distributed randomly in such a way that they stay close to the parent plant. This can be achieved by randomly distributing the seeds over the search space with mean equal to zero and variable variance. The standard deviation (SD), is given as;


where, it_max_ is the maximum number of iterations, S_it_ is the SD at the present time step, m is the nonlinear modulation index, S_i_ is the initial SD and S_f_ is the final SD. The produced seeds are then evaluated and allowed to go forward for further production if they provide better solutions than the parent weeds with lower fitness in a colony.

#### Competitive exclusion

The dispersed plants should go under competition so that only the best fit plants are kept for further generation. So, after some iteration, whenever the number of plants in a colony reaches the maximum, some of the unfit or less fit plants should be replaced by better fit plants. For this purpose, the parents and the offspring are ranked together and according to the fitness value some of the unfit candidates are excluded from the colony and thus keeping the final set of population equal to the maximum number of plants.

**Figure 3:**
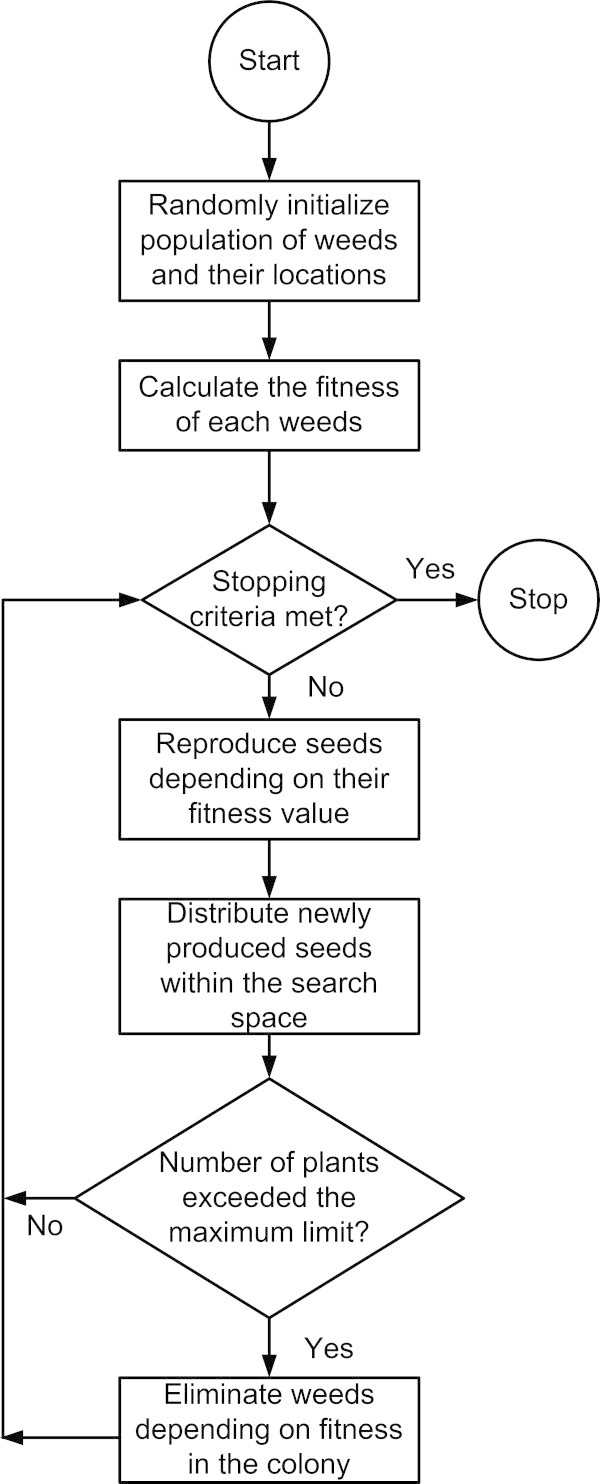
**Flowchart of IWO algorithm.**

The algorithm stops until the maximum number of iteration (it_max_) is reached or some predefined stopping criterion is fulfilled. In this work, the stopping criteria is set in such a way that if the fitness value does not change for the last 50 iterations, the algorithm would consider that it has obtained an optimal solution. The flowchart of the IWO algorithm is provided in Figure 3.

## Simulation results

The expectation from IWO tuned SSSC damping controller is to provide faster solution to power oscillation damping, quick settling time for the states and the overshoot/undershoot within acceptable limit. This will require some control effort. Again, as there is a hardware limit of designed controller system, for the case of SSSC, the control input parameter m_b_ should be within its limit. In this work, the boundaries for the modulation index are set between m_bmin_ and m_bmax_. The values of m_bmin_ and m_bmax_ are given in the Appendix.

For any power system, changes in operating conditions of system are common phenomenon. For a good design of damping controller, besides the maximum effectiveness of the controller, the robustness of damping controller to the variations of power system operating conditions is an equally important factor to be taken under consideration. Hence it is desirable for SSSC optimal POD controller to be able to tolerate changes in operating point and the performance should be satisfactory over a range of change of operating conditions. Therefore, it is particularly important to study the effect of load variations on the performance of designed controller. Keeping these in mind, to exhibit the effectiveness of designed POD controller, simulation studies has been carried out for three different operating conditions of the system which are listed in Table 1.

**Table 1:** **Various loading conditions**

Loading condition	P _e_(p.u)	V _t_(p.u)	Q _e_(p.u)
Light	0.8	1.0	0.0808
Nominal	1.0	1.0	0.127
Heavy	1.2	1.1	0.6068

The effectiveness of the optimization algorithm can be interpreted as i) the number of iterations taken by the optimizer to reach at the optimal solution, ii) the level of objective achieved in the form of minimization/maximization and iii) ability to search for an optimal result within the given constraints. The constraints are interpreted as the boundary values of the PI controller gains and the values are provided in the Appendix. In view of the above, the performance of IWO is compared with that of the Particle Swarm optimization (PSO) for the designing of the controller. Detail discussion on PSO can be found in (Kennedy & Eberhart 1995).

Numerical values of relevant parameters of the test system are provided in the Appendix. Parameters used for IWO and PSO optimizers are listed in Table 2.

**Table 2:** **IWO and PSO parameters**

IWO		PSO	
Parameter	Value	Parameter	Value
Population	30	Population	30
Number of generation	500	Number of generation	500
m	3	weight	0.9
S_i_	1	alpha	0.99
S_f_	0.0001		

Different simulation cases considered in this work are:

i) A mechanical torque pulse having a magnitude of 0.2 p.u. is applied at 1.0 sec and removed at 1.083 sec.
ii) A three phase short circuit fault is applied at the middle of one of the transmission line at 1.0 sec and the fault is cleared at 1.083 sec. So, the post fault and the pre fault transmission line reactance remain same.
iii) A three phase short circuit fault is applied at the middle of one of the transmission line at 1.0 sec and the faulted line is removed at 1.083 sec. So, the transmission line reactance gets doubled in the post fault period.

All of the above cases are simulated for the three different operating conditions provided in Table 1.

## Results and discussion

**Figure 4:**
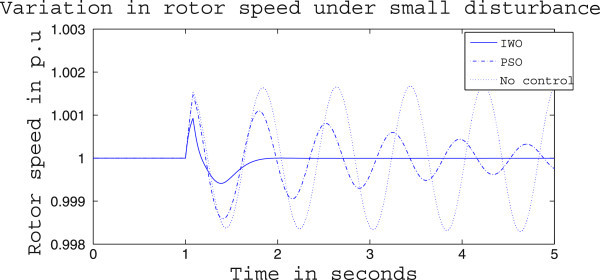
**Rotor speed response for case (i) under light load condition.**

**Figure 5:**
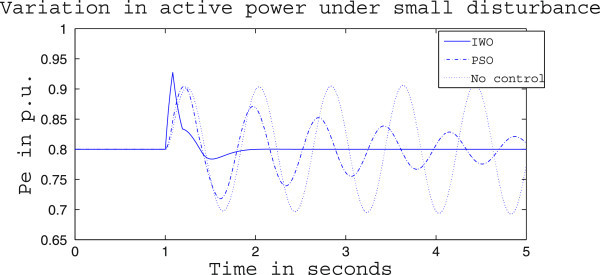
**Active power response for case (i) under light load condition.**

**Figure 6:**
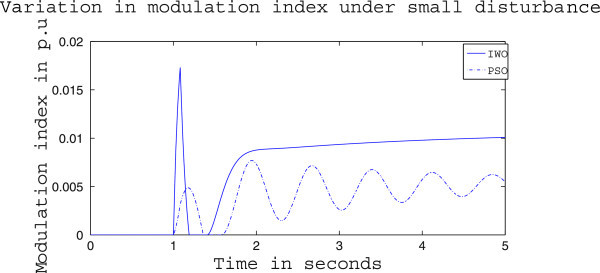
**Variation of control signal m**_**b**_ **for case (i) under light load condition.**

**Figure 7:**
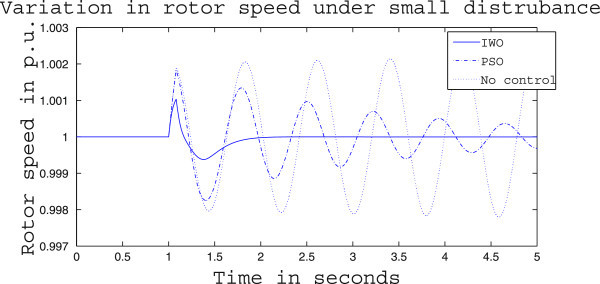
**Rotor speed response for case (i) under nominal load condition.**

**Figure 8:**
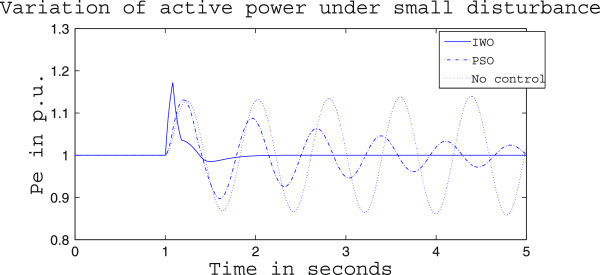
**Active power response for case (i) under nominal load condition.**

**Figure 9:**
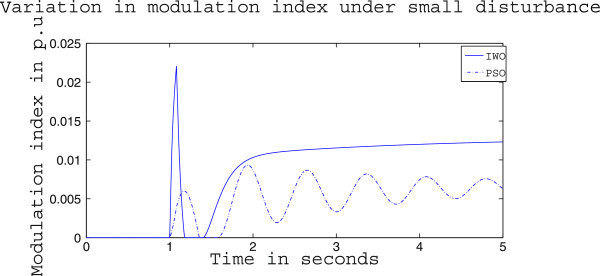
**Variation of control signal m**_**b**_ **for case (i) under nominal load condition.**

**Figure 10:**
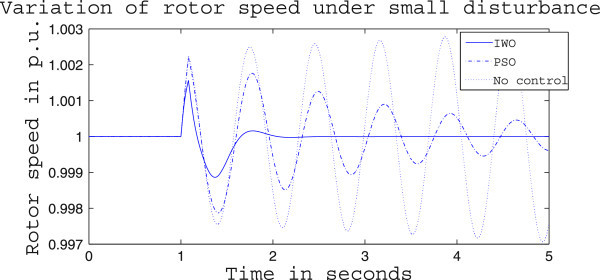
**Rotor speed response for case (i) under heavy load condition.**

**Figure 11:**
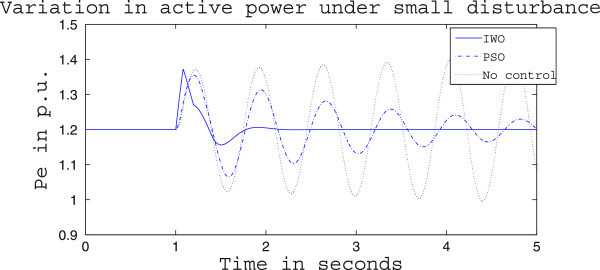
**Active power response for case (i) under heavy load condition.**

**Figure 12:**
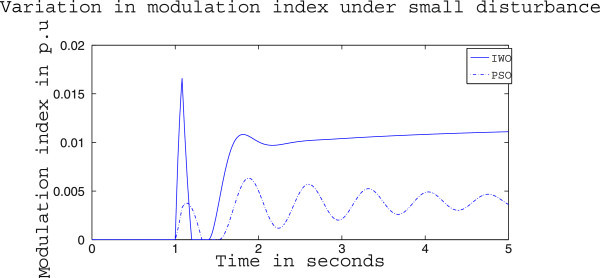
**Variation of control signal m**_**b**_ **for case (i) under heavy load condition.**

Figures 4, 5 and 6 presents the results of case (i) for light load conditions. It is seen that under no control the system experiences oscillatory instability as the rotor angle and active power oscillations are increasing with time. The introduction of optimized control action stabilizes the system for both PSO and IWO algorithms. But the damping action achieved is lot better for IWO than that of PSO. PSO optimized control action dampens the oscillation at a slow rate and even after the 5 sec simulation it requires some more time to reach to a steady value. On the other hand the IWO optimized control action can damp out the system oscillation within 1 sec of the introduction of the dynamics. Figure 6 shows that the control effort required by both algorithms are within the limit but the one with IWO settles to a steady value of 0.01 p.u. whereas the PSO control action keeps oscillating around 0.005 p.u.Figures 7, 8 and 9 presents the results of case (i) under nominal load condition. With increase in loading level the first swing of the rotor speed goes a little higher but the controller successfully damps out the subsequent oscillations. Here, too, the performance of the IWO optimizer is found superior than the PSO optimizer. Figure 9 ensures the fact that a little more control effort is required for the nominal load condition compared to the light load condition as the IWO optimized control signal settles at around 0.0125 p.u.Figures 10, 11 and 12 presents the results of case (i) under heavy load condition. Further increase in the first swing of rotor speed is noticed and the superiority of the IWO optimizer compared to that of PSO is again justified. The control effort requirement in Figure 12 changes ever so slightly compared to the nominal loading case in Figure 9.

**Figure 13:**
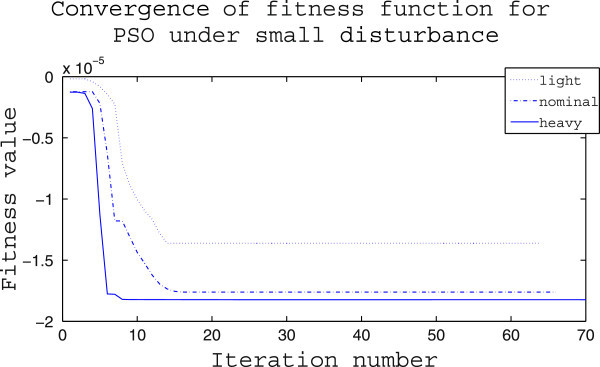
**Convergence of PSO optimizer for different loading in case (i).**

**Figure 14:**
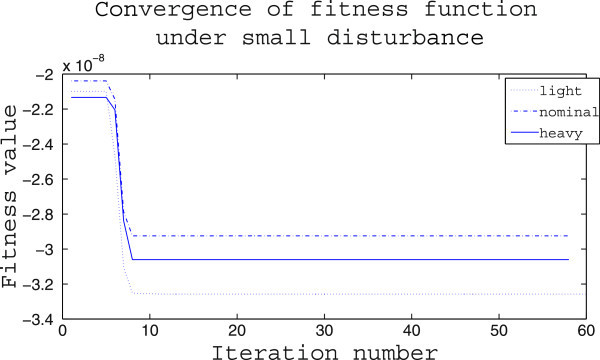
**Convergence of IWO optimizer for different loading in case (i).**

The supremacy of IWO optimizer over PSO optimizer is again tested in terms of their performance for converging to a minimum value of the fitness function and the results are provided in Figures 13, 14. Figure 13 shows that the minimization achieved by PSO is in the range of 10^−5^ and except in the heavy loading condition the number of iterations taken for convergence is larger than 10. In comparison, from Figure 14 it is seen that in all cases the algorithm converges within 10 iterations and the minimization achieved is in the order of 10^−8^.

**Figure 15:**
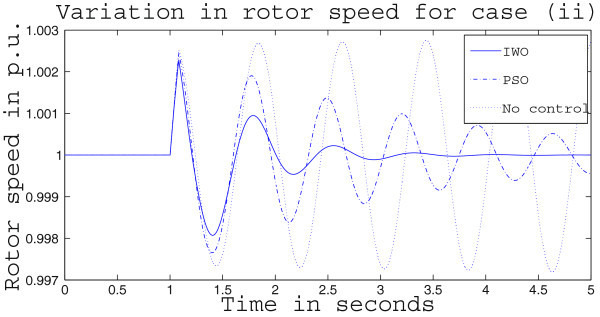
**Rotor speed response for case (ii) under light load condition.**

**Figure 16:**
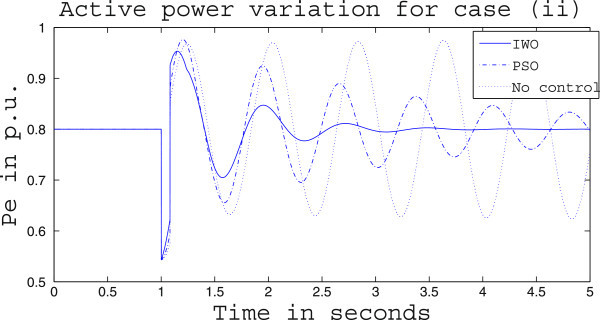
**Active power response for case (ii) under light load condition.**

**Figure 17:**
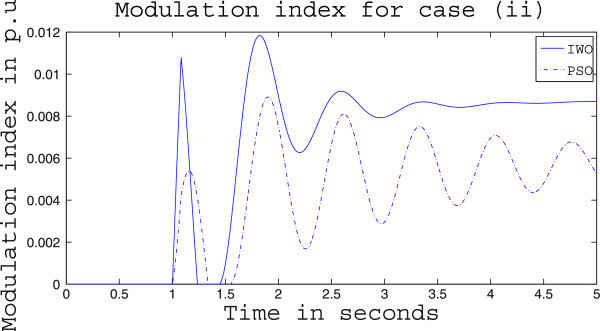
**Variation of control signal m**_**b**_ **for case (ii) under light load condition.**

**Figure 18:**
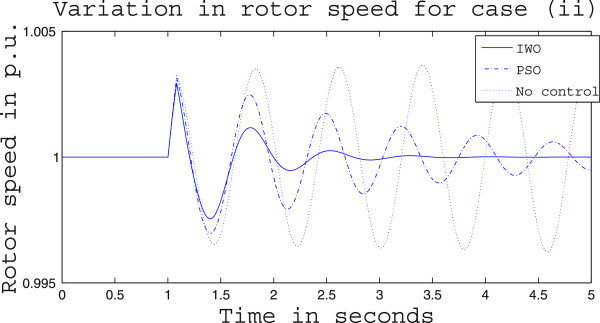
**Rotor speed response for case (ii) under nominal load condition.**

**Figure 19:**
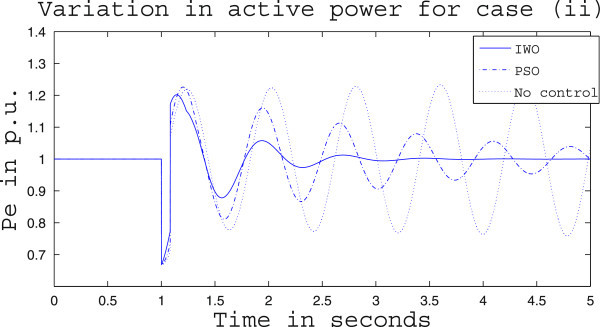
**Active power response for case (ii) under nominal load condition.**

**Figure 20:**
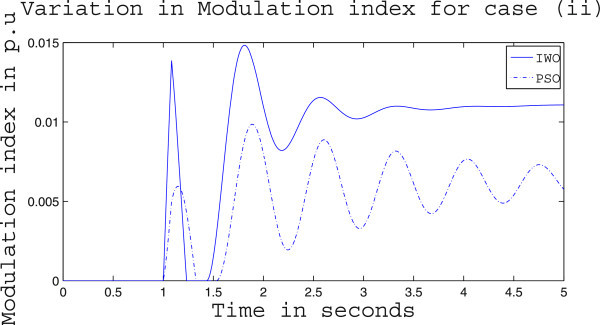
**Variation of control signal m**_**b**_ **for case (ii) under nominal load condition.**

**Figure 21:**
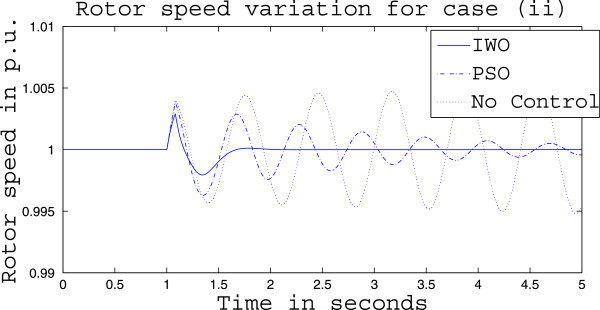
**Rotor speed response for case (ii) under heavy load condition.**

**Figure 22:**
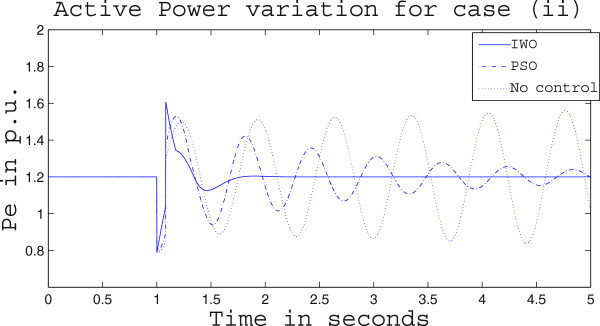
**Active power response for case (ii) under heavy load condition.**

**Figure 23:**
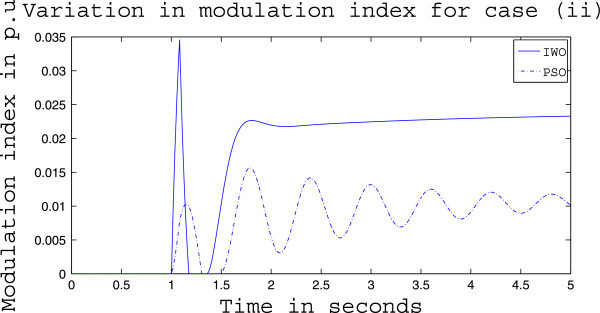
**Variation of control signal m**_**b**_ **for case (ii) under heavy load condition.**

**Figure 24:**
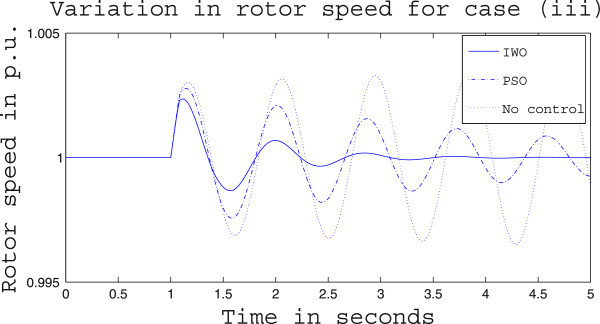
**Rotor speed response for case (iii) under light load condition.**

**Figure 25:**
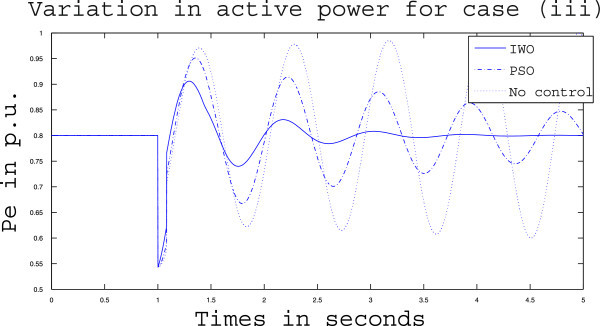
**Active power response for case (iii) under light load condition.**

**Figure 26:**
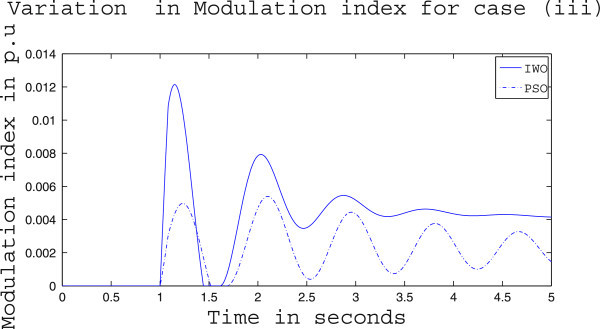
**Variation of control signal m**_**b**_ **for case (iii) under light load condition.**

**Figure 27:**
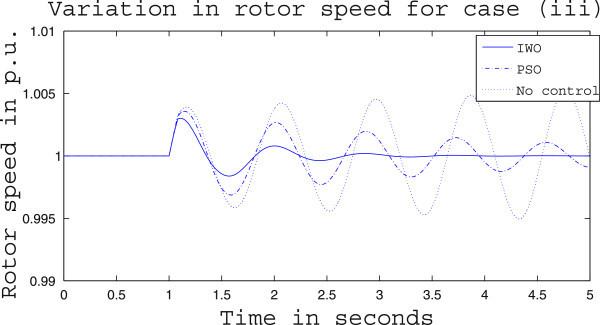
**Rotor speed response for case (iii) under nominal load condition.**

**Figure 28:**
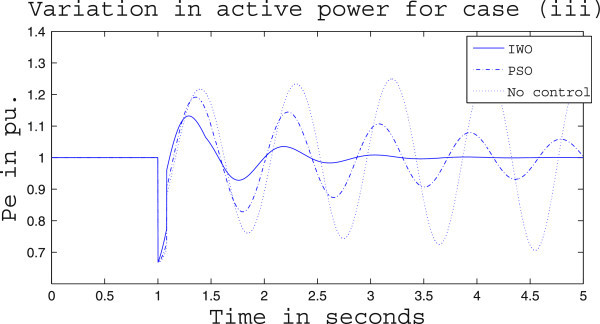
**Active power response for case (iii) under nominal load condition.**

**Figure 29:**
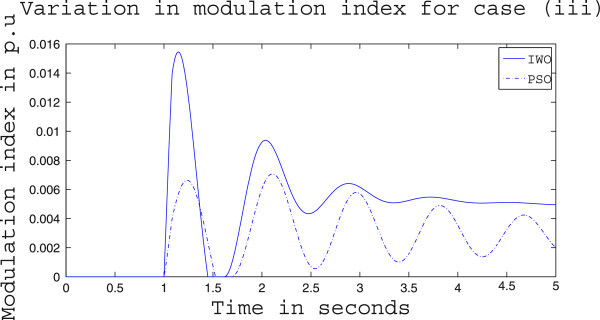
**Variation of control signal m**_**b**_ **for case (iii) under nominal load condition.**

**Figure 30:**
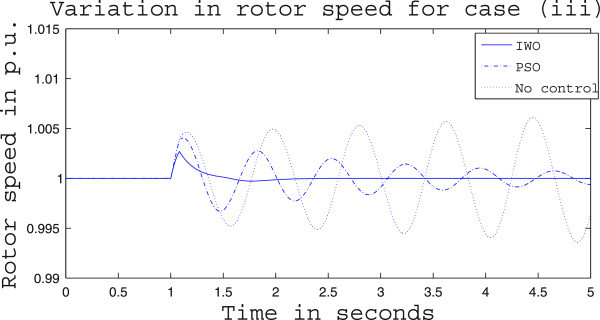
**Rotor speed response for case (iii) under heavy load condition.**

**Figure 31:**
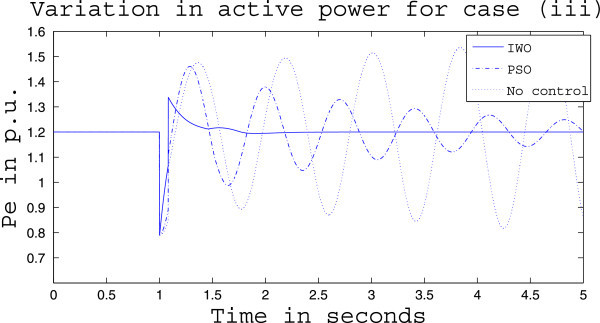
**Active power response for case (iii) under heavy load condition.**

**Figure 32:**
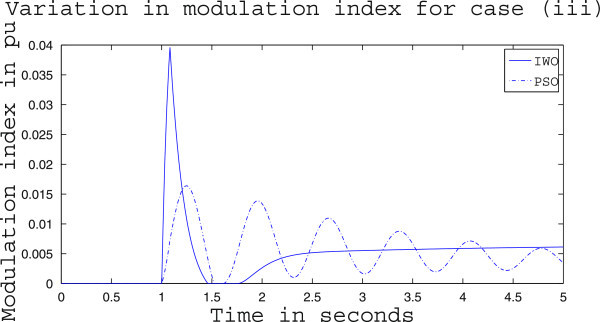
**Variation of control signal m**_**b**_ **for case (iii) under heavy load condition.**

Simulation results for case (ii) are presented in Figures 15, 16, 17, 18, 19, 20, 21, 22 and 23. From Figure 15 it is observed that under this case the rotor speed deviation from the synchronous speed continues for a little longer duration compared to case (i) but the IWO optimizer eventually dampens out the oscillation within 2.0 seconds of the arrival of the fault. The PSO optimizer takes much longer time to reduce the oscillation. Similar observation is found in Figure 16 for active power dynamics. Variation in the control signal m_b_ is presented in Figure 17 and it is clear that the IWO optimizer settles to a steady control signal much earlier than the PSO optimizer. Change in the operating condition from light to nominal or heavy does not change the outcome of the analysis as found from Figures 18, 19, 20, 21, 22 and 23. Even the change in fault scenario yields the same conclusion regarding the performance of IWO and PSO optimizer which can be observed from Figures 24, 25, 26, 27, 28, 29, 30, 31 and 32 for case (iii).

The plots of the control signal (m_b_) reveals the fact that in all cases both IWO and PSO can keep the value of the control signal within its maximum and minimum limits. The results of the optimized K_p_ and K_i_ parameters for IWO and PSO are presented in Table 3.

**Table 3:** **Optimized parameters of IWO and PSO for different case studies**

Optimizer	Case	Loading	K _p_	K _i_
PSO	I	Light	**1**	**28**
		Nominal	**1**	**28**
		Heavy	**1**	12.5119
	II	Light	**1**	17.9417
		Nominal	**1**	14.5325
		Heavy	**1**	25.4302
	III	Light	**1**	6.2319
		Nominal	**1**	6.6266
		Heavy	**1**	21.9208
IWO	I	Light	17.760	**28**
		Nominal	20.454	27.3489
		Heavy	9.8019	21.9525
	II	Light	4.3441	11.4455
		Nominal	4.3441	11.4455
		Heavy	11.4738	15
	III	Light	4.3441	11.4455
		Nominal	4.3441	11.4455
		Heavy	15	0.8943

The incidents where the optimizer hits the limit are shown in bolded form. It is found that the PSO optimizer reaches the lower limit of K_p_ in each case and the higher limit of K_i_ for the first two scenarios. On the other hand, the IWO optimizer hits the upper limit of K_i_ only in one scenario. This gives an indication of the better performance of IWO in finding an optimal solution within a confined search space.

## Conclusion

Power system stability enhancement using SSSC based damping controller is studied in this paper. A PI controller based design is considered where the optimal parameters of the controller are found by minimizing a time integral of absolute error based objective function. Different fault scenarios and loading conditions are studied for an SMIB system and IWO technique is employed to search for the optimal controller parameters. The performance of IWO optimizer is compared to that of PSO optimizer in a constrained search space. The simulation results show the superiority of IWO over PSO in tuning SSSC controller for the damping of power oscillations of a single machine power system.

## Appendix

Synchronous Generator: (2100 MVA, 13.8 kV).

f_b_ = 60 Hz, ω_0_ = 377 rad/sec, D = 0, M = 8.0 MJ/MVA, *x*_*d*_=1.0 p.u., , *x*_*q*_=0.6 p.u., .

Exciter: K_A_ = 10.0, T_A_ = 0.01 sec.

SSSC: S_nom_ = 100 MVA, V_noms_ = 500 kV (AC side), V_nomp_ = 5 kV (DC side), V_dcrated_ =2*V_nomp_, C_dc_ = 2.0 p.u., V_dc0_ = 10.0 p.u., m_b0_ = 0, *δ*_*b*_= − 30.88°, m_bmax_ = 1.0, m_bmin_ = 0.

Transformer: (2100 MVA, 13.8/500 kV).

X_t_ = 0.1 p.u.,

Transmission Line: X_L1_ = X_L2_ = 0.3 p.u.

Infinite Bus: V_b_ = 1.0 p.u.

PI Controller gain Limits: K_pmin_ = K_imin_ = 1, K_pmax_ = K_imax_ = 28.
